# Zn-doped Mono- and Biphasic Calcium Phosphate Materials Derived from Agriculture Waste and Their Potential Biomedical Applications: Part I

**DOI:** 10.3390/ma16051971

**Published:** 2023-02-28

**Authors:** Marta Kalbarczyk, Aleksandra Szcześ, Anna Belcarz, Paulina Kazimierczak, Zoltan May

**Affiliations:** 1 Department of Interfacial Phenomena, Institute of Chemical Sciences, Faculty of Chemistry, Maria Curie-Skłodowska University, 20-031 Lublin, Poland; 2 Department of Electrical Engineering, Lublin University of Technology, 20-618 Lublin, Poland; 3 Chair and Department of Biochemistry and Biotechnology, Medical University of Lublin, 20-093 Lublin, Poland; 4 Independent Unit of Tissue Engineering and Regenerative Medicine, Medical University of Lublin, 20-093 Lublin, Poland; 5 Plasma Chemistry Research Group, Institute of Materials and Environmental Sciences, Research Centre for Natural Sciences, Magyar Tudósok krt. 2, 1117 Budapest, Hungary

**Keywords:** hydroxyapatite, calcium phosphate, eggshells, Zn doping, antibacterial activity, cytotoxicity

## Abstract

In this study, calcium phosphate materials were obtained via a simple, eco-friendly wet synthesis method using hen eggshells as a calcium source. It was shown that Zn ions were successfully incorporated into hydroxyapatite (HA). The obtained ceramic composition depends on the zinc content. When doped with 10 mol % of Zn, in addition to HA and Zn-doped HA, DCPD (dicalcium phosphate dihydrate) appeared and its content increased with the increase in Zn concentration. All doped HA materials exhibited antimicrobial activity against *S. aureus* and *E. coli*. Nevertheless, fabricated samples significantly decreased preosteoblast (MC3T3-E1 Subclone 4) viability in vitro, exerting a cytotoxic effect which probably resulted from their high ionic reactivity.

## 1. Introduction

The increasing life expectancy requires the production of new bone and tooth replacement materials with certain properties. Calcium phosphate materials are recognized as excellent substitutes; among them is hydroxyapatite (HA) Ca_10_(PO_4_)_6_ (OH)_2_, which is the main component of mammalian bones and teeth [1]. As HA exhibits desirable properties for orthopedic implants, such as osteoconductivity, biocompatibility and bioactivity, research on its preparation and use has attracted attention [1,2,3,4,5,6,7,8,9,10,11,12,13,14,15,16,17]. The possibilities of using HA in implantology as ceramics [1,5,7,8,9,10,11,12,17,18,19], composite materials [3,20] and coatings on metal implants [16,18,21,22] are being studied. To obtain biomaterials with appropriate physicochemical properties, biphasic calcium phosphate (BCP) materials are also intensively investigated [23]. A mixture of HA and β-TCP (β-tricalcium phosphate) is the most commonly used ceramic material. Changing the HA/β-TCP ratio allows the modification of the solubility of ceramic, which is one of the most important parameters to control. Another important aspect that should be considered is microbial infection and biofilm formation prevention. Various antibacterial agents are inserted into bioceramics to reduce or eliminate such risks. *Staphylococcus aureus* and *Escherichia coli* are the bacterial strains most often indicated to cause post-implantation infections [8,22]. The popular method of introducing antibacterial properties is chemical modification by metal ion incorporation, such as Ag^+^, Zn^2+^, Cu^2+^ and Ce^3+^ [5,8,9,10,11,12,18,22]. Among them, Zn^2+^ ions have been intensively studied as a factor improving the antibacterial properties of biomaterials [5,8,9,11,12,18,22].

Thian et al. [5] demonstrated antimicrobial capability against *S. aureus* after contact with 1.6 wt% Zn-doped HA. In [8] *E. coli* and *S. aureus*, growth inhibition was demonstrated in contact with 5, 10, 15 and 20% Zn-doped Zn/HA scaffolds. The zone of inhibition increased with increasing zinc content. However, in the case of pure HA, no zone of inhibition was observed. Hidalgo-Robatto et al. [22] showed that HA coatings with incorporated 2.5% Zn and Cu reduced the growth of *E. coli* and *S. aureus* bacteria significantly compared to undoped HA, which promoted the growth of bacteria. Additionally, Sridevi et al. [18] proved the antibacterial activity of a Zn-HAP/CNC biocomposite coating against the same strains of bacteria, but reported a better performance against *E. coli*. On the contrary, in paper [16], better antibacterial efficacy was obtained against *S. aureus* than against *E. coli* using HA + Zn coatings with 5 and 10 mol% of Zn.

As shown in the previous study, it is possible to obtain biphasic calcium phosphate materials by a low-cost precipitation method using noncalcined eggshells as a calcium ion source [14,15]. The use of agricultural waste in the form of avian eggshells is extensively studied in the literature [14,15,18,19,24,25], also for economic reasons as it is a cost-effective method. Geng et al. [24] proved that the hydroxyapatite obtained using eggshells exhibited better biological performances compared to that obtained using Ca(NO_3_)_2_·4H_2_O. According to the authors, this may be related to the trace element content in eggshells, such as Mg. Additionally, these properties have been enhanced by doping with Sr^2+^ ions. Kim et al. [25] compared, in vivo, the new-bone-forming ability of commercially-available HA and biphasic powders and bioceramics derived from eggshells using a rabbit bone graft model. It was shown that in the early stages (4 weeks), the HA and β-TCP scaffolds obtained from eggshells promoted the formation of new bones to a greater extent.

The aim of this study is to determine the effect of doping with zinc ions on the composition, morphology, cytotoxicity and antimicrobial properties of calcium phosphate minerals obtained via this simple and eco-friendly wet method using hen eggshells.

## 2. Materials and Methods

Di-sodium hydrogen phosphate dodecahydrate (>99%), 65% nitric acid and 96% ethyl alcohol were purchased from Avantor (Gliwice, Poland), and zinc nitrate hexahydrate (ppa grade) was obtained from Chempur (Piekary Śląskie, Poland). All reagents were used without further purification.

The Milli-Q Plus system (Millipore, Burligton, MA, USA) was applied to purify the water for solution preparation, resulting in a water resistivity of 18.2 MΩcm.

### 2.1. The Synthesis Procedure

**Scheme 1:**
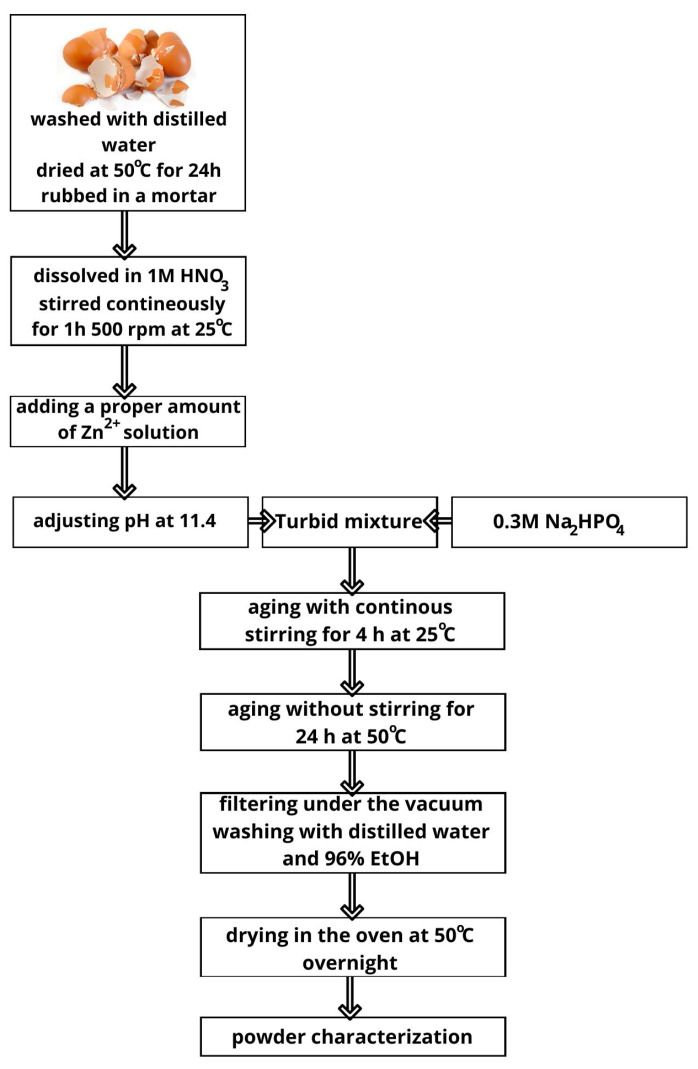
Synthesis scheme.

The hen eggshells collected from the residential neighborhood of Lublin were washed in distilled water and dried at 50 °C. The calculated number of eggshells were dissolved in 1 M nitric acid and stirred using a magnetic stirrer at 500 rpm for one hour. Then, the initial pH value was adjusted to 11.4 using a 1 M sodium hydroxide solution. Next, a 0.3 M Na_2_HPO_4_ solution was added dropwise to the obtained solution using a peristaltic pump with a flow rate of 20 mL/min and the suspensions were stirred for 4 h using a magnetic stirrer at 500 rpm at 25 °C. The stoichiometric molar Ca/P ratio was kept at 0.6 and the sample was denoted as 0% Zn. The calcium content in the eggshells was assumed on the basis of previous DTG studies to be 95% [14]. Then, the prepared mixtures were subjected to the aging process in an oven at 50 °C for 24 h, then filtered through vacuum filtration using membrane filters with a pore size equal to 0.45 µm, rinsed with Milli-Q water and ethanol and dried overnight at 50 °C (Scheme 1).

To introduce Zn ions to the material, after dissolving the eggshells, first, the proper amount of 0.3 M zinc nitrate solution was added dropwise to the beaker, the pH was adjusted at 11.4 and then, right away, a 0.3 M Na_2_HPO_4_ solution was added. The rest of the procedure remained unchanged. The planed molar content of Zn in HA was 5, 10, 15 and 20 molar % (later samples denoted as 5% Zn, 10% Zn, 15% Zn and 20% Zn). The stoichiometric planned molar ratio (Ca + Zn)/P was kept constant, i.e., 0.6.

For comparison in the cytotoxicity and antibacterial activity studies, the powder obtained in [15] was also used, using eggshells without the eggshell membranes (hereinafter referred to as 0% Zn(-EMS)).

### 2.2. The Obtained Materials’ Characterization

The Attenuated Total Reflectance Fourier Transform Infrared Spectroscopy spectra were performed with the spectrometer Nicolet IS 10 (Thermo Fisher Scientific, Waltham, MA, USA), whereas the X-ray diffractograms were conducted using a PANalytical Empyrean diffractometer (Malvern PANalytical, UK). Both techniques were employed to examine the samples’ composition. For XRD measurements, we applied the PIXcel-3D detector using monochromated Cu–Kα radiation (λ = 1.54184 Å), 40 kV and 35 mA, in the 2θ range of 4.1−60°, with an acquisition step of 0.02° and an acquisition time of 100 s. The qualitative and quantitative analyses were determined with the HighScore Plus 3.0e (3.0.5) software supplied by PANalytical with the ICDD PDF4 + 2019 diffraction database combined with the Rietveld method [26,27].

The samples’ morphology was determined by scanning electron microscopy (DualBeam Quanta 3D FEG, FEI, Hillsboro, OR, USA) with an Everhart–Thornley detector (ETD) (with an accelerating voltage of 30 kV, under a high-vacuum condition) and a high-resolution scanning transmission electron microscope (Titan3™ G2 60-300, FEI, Hillsboro, OR, USA) with an acceleration voltage range of 60 to 300 kV.

The Accelerated Surface Area and Porosimetry System ASAP 2420 (Micromeritics Inc., Norcross, GA, USA) allowed us to measure the low-temperature (−195.8 °C) nitrogen adsorption-desorption isotherms and calculate the porosimertic parameters as well as Brunauer–Emmett–Teller (BET) surface area. The Barrett–Joyner–Halenda (BJH) method based on the desorption branch of isotherms was applied to determine the pore size distribution [28]. All the samples were degassed at 200 °C.

The X-ray photoelectron spectroscopy (XPS) studies were carried out using the multi-chamber UHV system (PREVAC, Poland). Spectra were collected by means of the hemispherical Scienta R4000 electron analyzer. The Scienta SAX-100 X-ray source (Al Kα, 1486.6 eV, 0.8 eV band) equipped with the XM 650 X-Ray Monochromator (0.2 eV band) was used as complementary equipment.

The pass energy of the analyzer was set to 200 eV for the survey spectra (with 500 meV step), and 50 eV for the regions (high resolution spectra): C 1 s, O 1 s, S 2p and Cu 2p (with 50 meV step). The base pressure in the analysis chamber was 5·10-9 mbar. During the spectra collection, it was not higher than 3·10^−8^ mbar.

Elemental analysis of doped Zn ions was conducted using inductively coupled plasma optical emission spectroscopy spectrometry ICP-OES (Spectro Genesis, Kleve, Germany) after dissolving the obtained powders in boiling nitric acid.

### 2.3. Antibacterial Activity Studies

#### 2.3.1. Strains and Maintenance

*Staphylococcus aureus* ATCC 25923 and *Escherichia coli* ATCC 25922 reference bacterial strains were used in experiment. Both bacteria were maintained in microbanks at −80 °C, then cultured in Mueller–Hinton (M–H) agar medium (Biomaxima, Poland) at 37 °C for 20–24 h and finally transferred into Mueller–Hinton (M–H) broth (Biomaxima, Poland) for a further 24 h incubation at 37 °C. Directly before the experiment, bacterial inoculates were obtained by dilution of the suspension to the titers selected individually for each strain.

Tested biomaterials were sterilized by EthO (ethylene oxide) before the test, which was performed in triplicate.

#### 2.3.2. Direct Contact Test

The test was performed as described earlier [2]. Briefly, sterilized hydroxyapatite powders were suspended in sterile PBS pH 7.4 to obtain suspensions of concentrations 2 mg/mL and 0.2 mg/mL, which were sonicated (Sonic-6, Polsonic, Warsaw, Poland) in ice-cold water for 90 min to obtain full dispersion. Bacterial working suspensions in M–H broth diluted 125-fold in sterile 0.9% NaCl were prepared for each strain: 6.0 × 10^5^ CFU/ mL for *S. aureus* and 6.0 × 10^3^ CFU/mL for *E. coli*. Then, powder suspensions and bacterial working solutions were mixed in sterile 5 mL screwed tubes in a proportion of 1:1, yielding the mixtures of 1 mg/mL or 0.1 mg/mL of powder, 3.0 × 10^5^ CFU/mL (for *S. aureus*) or 3.0 × 10^3^ CFU/mL (for *E. coli*) and M–H broth diluted 250-fold in sterile 0.9% NaCl. Positive controls for each bacterial strain were prepared by mixing PBS pH 7.4 and bacterial working solutions in the same proportions as for powder suspensions. Afterwards, the tubes were incubated at 37 °C, 24 h, at 20 rpm on an RM 5-30V CAT roller mixer (Ingenieurbüro CAT M.Zipperer, Ballrechten-Dottingen, Germany). Finally, 50 µL aliquots of the mixtures were plated onto M–H agar Petri dishes using an EasySpiral Dilute (Interscience, Saint-Nom-la-Bretèche, France) automatic plater (each sample in triplicate; plated in standard exponential mode). M–H agar plates were incubated at 37 °C for 18–28 h, depending on the strain. CFUs were then counted for each plate using a Scan 300 colony counter (Interscience, Saint-Nom-la-Bretèche, France).

### 2.4. Cytotoxicity Assessment In Vitro

The cytotoxicity assessment of fabricated calcium phosphate powders was performed according to the ISO 10993-5 standard by using 24 h extracts of the samples prepared in accordance with the ISO 10993-12 standard (maintaining the ratio of 100 mg sample per 1 mL complete culture medium). Mouse calvarial preosteoblast cell line (MC3T3-E1 Subclone 4, ATCC-LGC standards, Middlesex, Teddington, UK) was used in the cytotoxicity evaluation in vitro. The MC3T3-E1 cells were maintained in an alpha-MEM medium (Gibco, Life Technologies, Carlsbad, CA, USA) supplemented with 10% fetal bovine serum (Pan-Biotech GmbH, Aidenbach, Germany) and a penicillin–streptomycin solution (Sigma-Aldrich Chemicals, Warsaw, Poland) and cultured at 37 °C in a humidified atmosphere with 5% CO_2_. The cytotoxicity assessment of the fabricated calcium phosphate materials was evaluated by MTT assay (Sigma-Aldrich Chemicals, Warsaw, Poland). Briefly, the MC3T3-E1 cells were seeded into 96-well plates with a density of 2 × 10^4^ cells per well. After 24 h, the culture medium was discarded and replaced by 100 µL of appropriate samples extracts. The culture medium incubated without fabricated calcium phosphate materials served as the negative control of cytotoxicity (marked as control PS). The cells were incubated for a further 24 h. Next, the cell viability was evaluated using MTT assay. The obtained results of the MTT test were shown as the percentage of absorbance value obtained with the negative control. Additionally, the evaluation of Ca^2+^ and HPO_4_^2−^ in 24 h extracts of the samples was performed using colorimetric detection kits (BioMaxima, Lublin, Poland), according to the manufacturer’s instructions.

### 2.5. Statistical Analysis

The data were displayed as mean values ± SD. Tests were carried out at least in triplicate (*n* = 3). Statistically significant results between tested samples were considered at *p* < 0.05 and were determined using one-way ANOVA followed by Tukey’s test (GraphPad Prism 8.0.0 Software).

## 3. Results

### 3.1. ATR-FTIR Characterization of Undoped and Zn-Doped Powders

**Figure 1:**
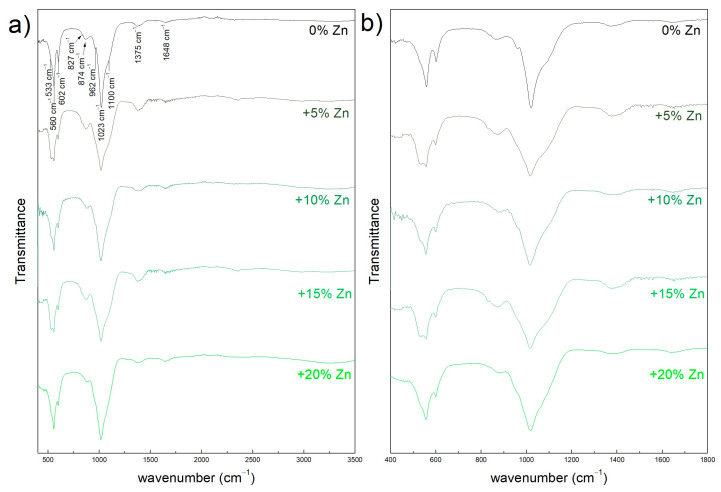
FTIR spectra of the obtained materials: without Zn (0% Zn) and with 5, 10 and 15 molar % of Zn ranging from: (**a**) 400–3500 cm^−1^, (**b**) 400–1800 cm^−1^.

Analysis of the composition of the obtained materials is the first step of research. Powders were obtained as a result of the synthesis. However, the material with the highest zinc content (20% Zn) was very hard and compact and was not suitable for FTIR ATR analysis. The FTIR spectra of all obtained materials (Figure 1) showed peaks characteristic of calcium phosphate materials: stretching mode of P-O bending at 962, 1032 and 1100 cm^−1^ and the peaks at 560 and 602 cm^−1^ assigned to the bending modes of O-P-O in the phosphate group [3,4,5]. The origin of the band at 827 cm^−1^ can be linked with the hydroxyl groups’ substitution by carbonate groups on the surface during crystal growth (A-type apatite), whereas the peaks at the range from 1300 to 1650 cm^−1^ are linked to the vibration of ${\mathrm{CO}}_{3}^{2-}$ related to the substitution of ${\mathrm{PO}}_{4}^{3-}$ (A-type apatite), as the result of the reaction between HA and carbon dioxide from the air as well as synthesis precursors [3,5,6]. The weak signal at the band position of 2363 cm^−1^ corresponds to the adsorbed ${\mathrm{CO}}_{3}^{2-}$ [29]. The wide, small band at 1375 cm^−1^ can be also associated with the residual ${\mathrm{NO}}_{3}^{-}$ groups from the reacting mixture [4].

The shape of the spectra indicates the multiphasic composition of all samples. The signal at 962 cm^−1^ is present only for the sample without Zn and origins from the β-TCP phase [7,29]. The band placed at c.a. 530, 877 cm^−1^, as well as bands at 1077, 1107 and 1125 cm^−1^ can be assigned to ${\mathrm{HPO}}_{4}^{2-}$ groups [30,31]. The last three mentioned peaks are visible as shoulders in the range from 1000 to 1200 cm^−1^, the intensity of which increases with the increase in Zn content in the tested materials. The presence of ${\mathrm{HPO}}_{4}^{2-}$ groups is typical for DCPD. Their presence in the sample may also indicate calcium-deficient apatite formation. It can be seen that the enhancement of the DPCD signal with the Zn content increase can be noticed, while the band originated from β-TCP disappears.

### 3.2. XRD, SEM, TEM and Porous Structure Characterization of Undoped and Zn-Doped Powders

**Figure 2:**
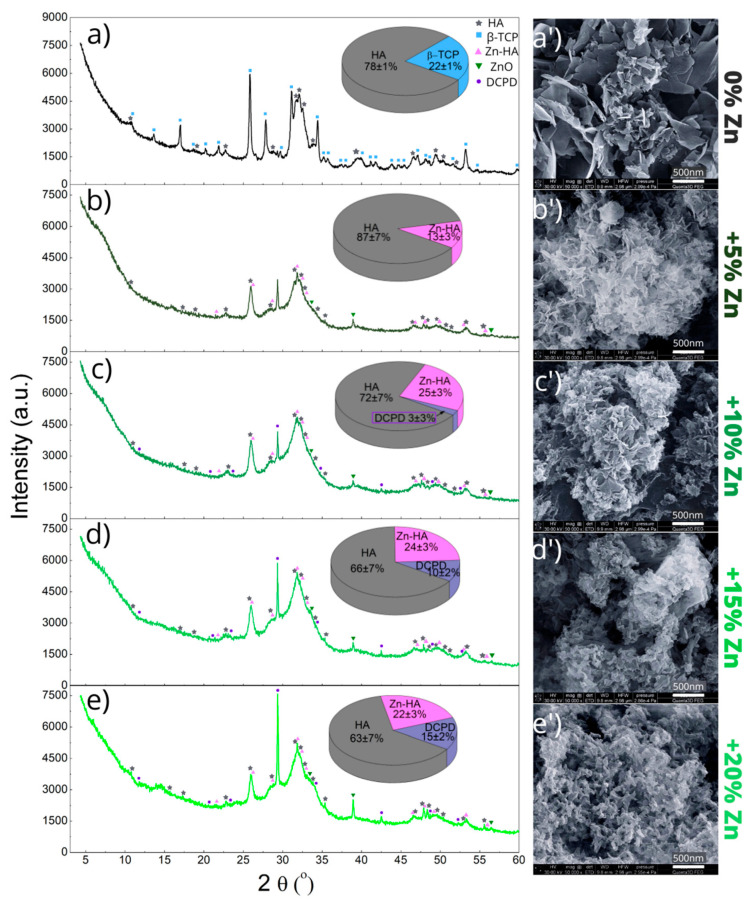
The XRD patterns (**a**–**e**) of the obtained products without and with the initial 5%, 10%, 15% and 20 molar %, respectively, and the SEM pictures of the samples (**a′**–**e′**).

To confirm the FTIR results, XRD studies were performed and the patterns are shown in Figure 2a–e. The composition of the products calculated with respect to Rietveld’s method [26] are attached with the patterns. The product synthesized without zinc additives was composed of 78% HA and 22% β-TCP. The presence of Zn highly affected the composition of the product. Doping with zinc ions caused the disappearance of β-TCP and the appearance of Zn-doped HA in the samples. It can be seen that the sample denoted as 5% Zn consisted of 87% HA and 13% Zn-HA. The further increase in the amount of Zn resulted in the presence of DCPD and the increase in its content in the products from 3% for the sample with 10 molar % of Zn up to 15 % for the highest Zn content. The increase in DCPD content was accompanied by a gradual decrease in HA content, with slightly changing Zn-HA content. It seems that a small amount of Zn can transform the less stable forms of calcium phosphate into HA and Zn-HA, while increasing the zinc ion content can stabilize the DCPD. This is in agreement with the literature data, showing the ability of zinc to inhibit the formation of HA and reduce the crystallite size [3,13].

The morphological changes in the samples are seen in Figure 2a′–e′. It can be seen that the sample without Zn additive is composed of large, flake-like aggregated structures. Even the smallest zinc ion content strongly affected the crystals’ shape and size. In the structure of the product with 5% initial Zn content, fine, irregular flakes of smaller size all stuck together are noticeable. Particle size decreased with increasing Zn content in the materials. However, further Zn content increases resulted in the formation of agglomerates with a cauliflower shape, where the crystals merged with each other. In the case of samples with an initial zinc content above 10%, the structure of the material became more compact. Changes in the morphology of the obtained materials were reflected in the values of the specific surface area as well as the size and volume of pores, as shown in Table 1 and Figure S1. Replacing 5 mole % of Ca with zinc ions caused the BET surface area to increase from 36.3 m^2^/g to 80.1 m^2^/g. With a further increase in the Zn content, the surface area gradually decreased to 56.0, 30.0 and 25.2 m^2^/g, respectively, with an initial Zn of 10%, 15% and 20%. Total pore volume, micropore volume, pore diameter and pore size distribution were subject to similar changes. It seems that the merging of particles into agglomerates eliminates the positive effect of increasing the surface area and pore volume, associated with the formation of smaller crystallites in the presence of Zn.

**Figure 3:**
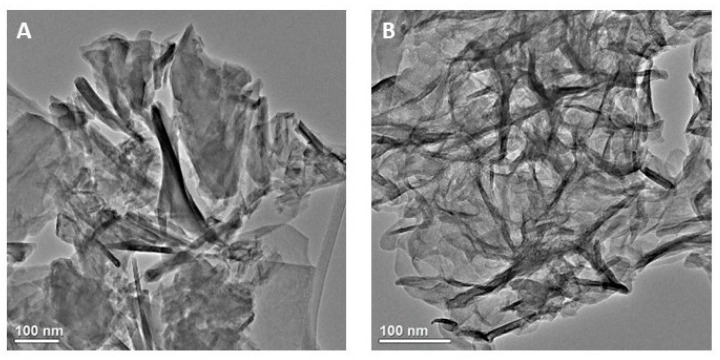
TEM images of the samples: (**A**) 0% Zn and (**B**) 10% Zn.

The morphology and composition of the samples without Zn and those initially doped with 10 % Zn ions were also characterized by TEM. It confirms the formation of aggregates consisting mostly of flake-like particles and a few rod-like ones in the absence of Zn (0% Zn). It is clearly seen that the presence of zinc mostly causes the formation of smaller, around 100 nm-long, rod-shaped crystals with a much smaller number of flake-like crystals (Figure 3). The EDX profile confirms the even distribution of Ca, O and P atoms, as well as in the case of doping with zinc ions, in the presence of Zn atoms (Figure S2).

### 3.3. ICP-OES Determination of Zn Content in the Doped Powders

**Figure 4:**
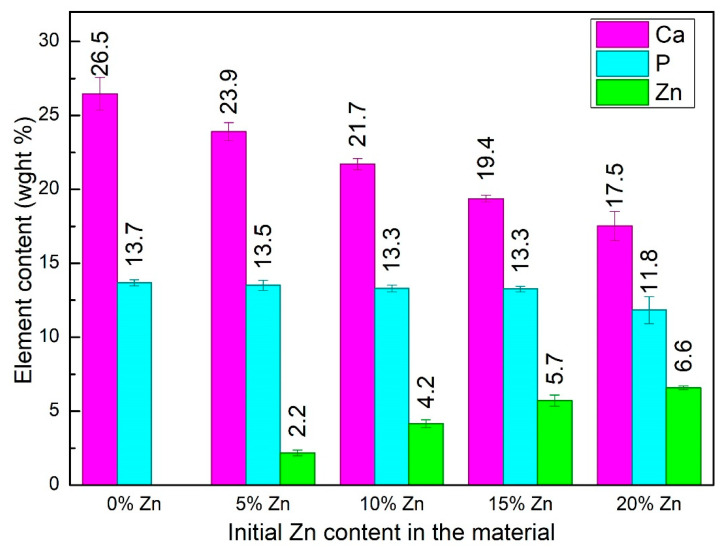
Zn, Ca and P content in the obtained materials.

The Zn content in the obtained materials determined using the ICP-OES technique is presented in Figure 4. It can be seen that the Zn content in the samples increased with the increase in the initial number of zinc ions, and they substituted an equivalent number of Ca ions, indicating good match between planned and experimental values (Table 2). The experimental Ca/P and (Ca + Zn)/P ratio was around 1.5 and decreased with increasing zinc ion content. This may be due to the formation of biphasic materials with different Ca to P ratios as well as defects in the crystal lattice. It is known from the literature that often, natural apatites are non-stoichiometric [3,4,23].

Despite the use of a Ca/P molar ratio of 0.6, the dominant form of the obtained calcium phosphates was hydroxyapatite, for which the Ca/P ratio is 1.67. The coexistence of more soluble forms with a lower Ca/P molar ratio, i.e., β-TCP and DCPD, may indicate that in the first stage of the synthesis, those forms were formed, of which the closest to the ratio of 0.6 is DCPD. However, it should be noted that at the alkaline pH of the synthesis, the stable form is hydroxyapatite. It can, therefore, be assumed that as a result of the aging process, the more soluble forms of calcium phosphates were transformed into HA, which is more stable in such an environment.

### 3.4. XPS Characterization of Undoped and 10% Zn-Doped Material

**Figure 5:**
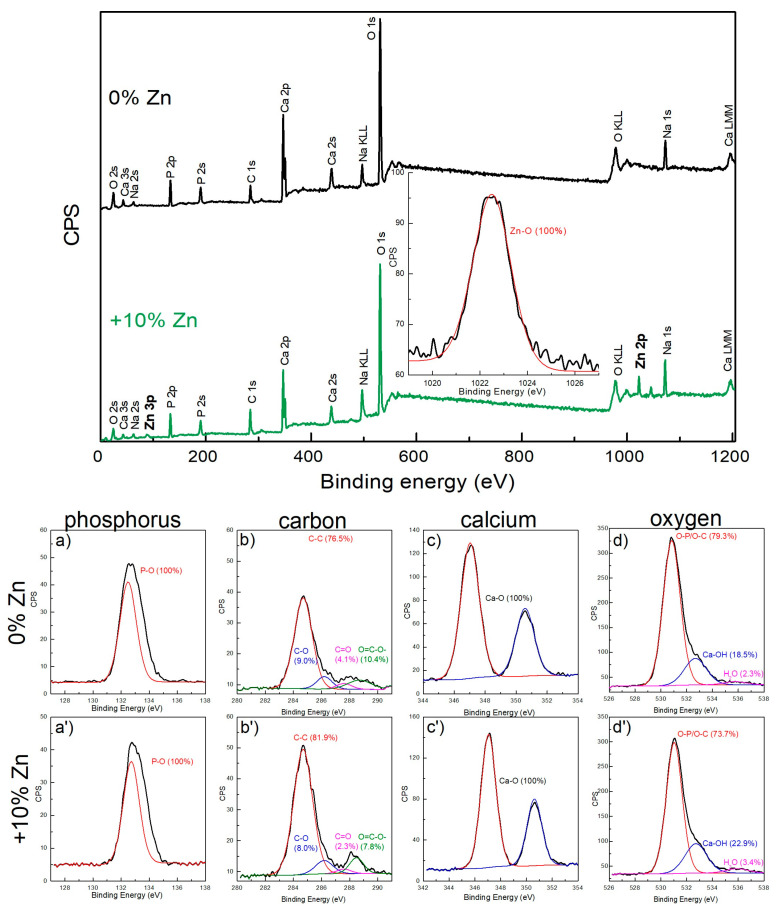
The XPS results along with the (**a**,**a′**) P2p, (**b**,**b′**) C1s, (**c**,**c′**) Ca2p and (**d**,**d′**) O1s XPS peaks in the samples (**a**–**d**) without (0% Zn) and (**a′**–**d′**) with 10 molar % of Zinc in the precursors (+10%Zn).

In the XPS spectra of both precipitates, the peak positions at the binding energies (BEs) characteristic for such elements as O, Ca, Na, P and C were noticed (Figure 5). Moreover, in the sample with 10 molar % of Zn, Zn-O binding was present (Figure 5), confirming the effective incorporation of Zn into the HA structure. All BE values were in good agreement with the literature data. The signals from P 2p, C 1s, Ca 2p and O 1s were analyzed in more detail to determine the binding character (Figure 5). It can be stated that phosphorus on the material surface was attached to the oxygen atoms completely, which was also confirmed in the O 1s spectra region at 531.0 eV in both samples and originated from phosphate groups [32]. The peaks in the range from 282–290 eV, as well as 526–538 eV, are complex. The first one can be considered a result of the overlapping of four peaks of C 1s: 284.7, 286.2, 287.6 and 288.5 eV, corresponding to C-C, C-O, C=O and O=C-O- groups, respectively [8,32,33]. It proves the carbonate groups’ presence on the material’s surface, whereas the C-C bond indicates the organic contaminations being a part of eggshells. Meanwhile, the second complex peak, deriving from O 1s, combines three components at 531.0, 532.7 and 535.9 eV, corresponding to O-P/O-C, Ca-O and H_2_O, respectively [32,33]. It confirms the presence of phosphates and a carbonate group and the binding between oxygen and calcium in the calcium phosphate structure, as well as hydroxyl bonds on the material surface, which is strongly related with calcium phosphate structure and directly indicates the HA and other forms of CPs detected with the XRD patterns. Moreover, the one-component signal from Zn 2p may indicate the substitution of calcium cations in the structure.

A Ca 2p signal is present in the form of doublet at 346.8 and 350.4 eV, attributed to Ca 2p_3/2_ and Ca 2p_1/2_. Both signals originate from a Ca-O bond; however, it indicates two forms of calcium ions linked with oxygen: the screw-axis Ca and columnar-axis Ca, surrounded by nine or seven oxygen atoms [3].

### 3.5. Antibacterial Activity of Undoped and Zn-Doped Materials

**Figure 6:**
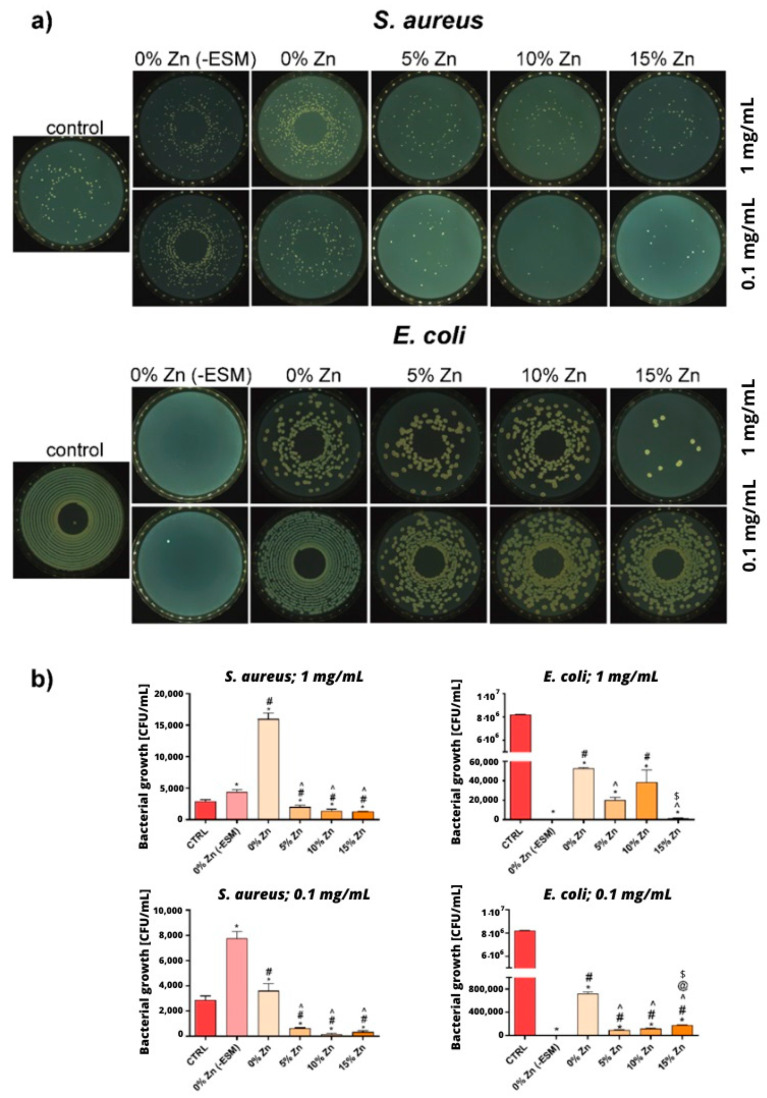
Results of bacterial growth in direct contact with tested powders presented as (**a**) plate images and (**b**) figures. (*) indicates statistically significant differences between the growth control (CTRL) and other samples, (#) indicates statistically significant results between 0% ZnO (-ESM) sample and other samples, (^) indicates statistically significant results between 0% ZnO sample and the other samples, (@) indicates statistically significant results between 5% ZnO sample and other samples, ($) indicates statistically significant results between 10% ZnO sample and other samples, according to one-way ANOVA with post hoc Tukey’s test (*p* < 0.05).

Taking into account the potential biomedical application of the obtained materials, the evaluation of antibacterial activity of the synthesized nanohydroxyapatite powders was conducted against the two bacterial strains that most commonly cause infection in humans, *E. coli* (Gram negative) and *S. aureus* (Gramm positive) (Figure 6). Their antibacterial activity was expected, in light of earlier reports [8,9,10]. In direct contact with the tested powders, the bacteria used in this study reacted in different ways, depending on the sample. The viability of *S. aureus* was stimulated by contact with 1 mg/mL 0% Zn in comparison with the control (Figure 6). This phenomenon may be related to the abundant release of calcium and phosphate ions from hydroxyapatite powders and the content of organic compounds. It is known that calcium and phosphate ions are essential for the metabolism and growth of living cells, including bacteria. This hypothesis seems to be supported by the effect of hydroxyapatite deprived from organic compounds, namely 0% Zn (−ESM), which stimulated the viability of *S. aureus* to a lesser extent (3.6-fold) than 1 mg/mL 0% Zn powder (Figure 6a). For comparison, the material obtained in the previous work from chicken eggshells without eggshell membranes (0% Zn (−ESM)) was used [15]. The lack of antibacterial activity of HA against *S. aureus* can be found in the literature [5]. Hidalgo-Robatto et al. [22] also demonstrated the promotion of growth in *E. coli* and *S. aureus* bacteria in contact with HA coatings.

However, all powders containing Zn reduced the number of live bacteria, both in comparison with 0% Zn and with the control. In a lower concentration (0.1 mg/mL), all powders containing Zn also showed a significant bacterial-killing activity, while the 0% Zn reference powder did not exert a bacterial-growth-stimulating effect. Among Zn-doped powders, 10% Zn and 15% Zn were more effective as antibacterial agents than 5% Zn powder.

In contrast, the viability of *E. coli* was reduced by 1 mg/mL 0% Zn powder in comparison with the control (Figure 6). The presence of Zn (5–15%) enhanced the antibacterial effect of the powders, wherein this effect was the most significant for the sample with 15 initial molar % of Zn. For the powders in 0.1 mg/mL concentrations, the *E. coli* viability-reducing effect was less striking in the case of all samples, although Zn-containing powders were more effective than the 0% Zn powder. Additionally, in case of this bacterial strain, incubation with hydroxyapatite deprived of an organic compound (0% Zn (-ESM)) caused a reduction in the cells’ viability. This reduction was very effective, causing a complete reduction in viable *E. coli* cells in a 1 mg/mL concentration and almost complete reduction in a 0.1 mg/mL concentration. This phenomenon is in agreement with other reports. It was reported that hydroxyapatite was nontoxic for bacteria [5,13,22], and some observations suggested the antibacterial activity of pure nanohydroxyapatite, both artificial, such as nanoHA (obtained by the wet precipitation method or by the microwave-assisted method) [1,11], and natural source-derived [12]. Therefore, our results confirm the previous reports, in relation to *E. coli*.

### 3.6. Cytotoxicity Assessment In Vitro

The cytotoxicity assessment showed that fabricated powders significantly decreased MC3T3-E1 preosteoblasts’ viability compared to the control medium (Table 3). It is worth noting that according to ISO 10993-5, the reduction in cell viability below 70% caused by 100% biomaterial extract should be considered a cytotoxic effect. In this study, a decrease in cell viability was observed with increasing Zn content in materials (i.e., extracts of 5% Zn, 10% Zn and 15% Zn reduced cell viability to 47.6, 32.7 and 7.3%, respectively). Interestingly, the extract of 0% Zn material exhibited the highest toxic effect (cell viability was 6.4%). Thus, it may be concluded that the cytotoxicity of the fabricated powders was not only caused by the zinc content but also could have resulted from the remaining inorganic chemical compounds during the synthesis of calcium phosphate materials such as nitrate groups. It also must be noticed that bioceramics, unsintered or sintered at low temperatures, exhibit high ion reactivity, changing their ionic composition in the medium and thereby reducing cell viability [34,35]. Therefore, Ca^2+^ and HPO_4_^2−^ ion concentrations in extracts were evaluated (Table 3). The 0% Zn powder showed the highest uptake of Ca^2+^ ions compared to the other samples. In turn, all tested materials released a large number of HPO_4_^2−^ ions into the culture medium. The observed high ion reactivity of the fabricated powders may result from ion uptake, ionic substitutions or their dissolution [34,36]. Thus, in this study, it may be suggested that the observed cytotoxicity was probably related to zinc content, ion reactivity or remaining inorganic toxic compounds in materials during their synthesis. Nevertheless, further studies are needed. In turn, in another study by Jahangir et al. [12], sintered eggshells decreased cell viability to 5%. The authors suggested that calcium oxide was responsible for the high toxicity.

## 4. Conclusions

Zn-doped biphasic calcium phosphate materials were prepared using a simple wet precipitation method and the hen eggshells as a calcium source. Zn-undoped material consisted of 78% HA and 22% β-TCP. Zn doping resulted in the formation of zinc-substituted HA, which increased with the increase in zinc content. When the zinc content was equal to or greater than 10%, an additional form of calcium phosphate appeared, i.e., DCPD. The DCPD content depends on the amount of zinc and was 3%, 10% and 15% for samples doped with 10%, 15% and 20% Zn, respectively. The presence of Zn also affects the surface properties and structure of the obtained materials. The presence of 5% and 10% Zn increased the specific surface area from 36.3 m^2^/g to 80.1 and 56.5 m^2^/g, respectively. A further increase in the zinc content caused a decrease in the specific surface area, possibly through the formation of particle agglomerates. All Zn-doped materials exhibited antimicrobial activity against *E. coli* and *S. aureus*, which increased with the Zn content. Additionally, pure HA powder exhibited antimicrobial activity against *E. coli*, while promoting the growth of *S. aureus* regardless of the concentration of the powder. Nevertheless, the fabricated powders significantly decreased cell viability in vitro, which probably results from either zinc content or their high ionic reactivity resulting from the presence of the more soluble DPPC. It may also be suggested that the observed cytotoxicity is related to the remaining inorganic toxic compounds (e.g., nitrate groups) in the materials during their synthesis. In the future, it is worth focusing on the modification of the developed synthesis method of biphasic calcium phosphates to overcome cytotoxicity, increasing the chance for their biomedical applications.

Therefore, further research is planned to improve the biocompatibility of the obtained materials. In addition, taking into account the well-developed surface area, in the paper that follows, the adsorptive properties of the tested materials will be shown.

## Figures and Tables

**Table 1:** The porosimetric parameters of the obtained products.

	0% Zn	5% Zn	10% Zn	15% Zn	20% Zn
Specific surface area BET [m^2^/g]	36.3 ± 0.2	80.1 ± 0.6	56.0 ± 0.3	30.0 ± 0.2	25.2 ± 1.0
Total pore volume [cm^3^/g]	0.187	0.553	0.352	0.242	0.179
Micropore volume [cm^3^/g]	0.005	19.719	0.005	0.004	0.002
Average pore width (BET) [nm]	20.6	27.3	25.1	32.2	28.1
Pore diameter from the adsorption BJH [nm]	25.4	34.6	29.4	38.0	32.2

**Table 2:** Planned and experimentally obtained composition of the synthesized materials.

Sample	Zn Composition, mol %		(Ca + Zn)/P Molar Ratio
	Planned	Experimental	Experimental
0% Zn	0	0	1.50 ± 0.04
5% Zn	5	5.4 ± 0.3	1.45 ± 0.03
10% Zn	10	10.8 ± 0.6	1.42 ± 0.01
15% Zn	15	15.6 ± 0.9	1.34 ± 0.02
20% Zn	20	18.6 ± 1.0	1.42 ± 0.06

**Table 3:** Cytotoxicity evaluation of 24 h sample extracts against mouse calvarial preosteoblasts (MC3T3-E1 Subclone 4) by MTT assay and assessment of Ca^2+^ and HPO_4_^2−^ ion concentrations in extracts (PS control: the culture medium incubated in polystyrene without samples).

	PS Control	0%Zn (−ESM)	0% Zn	5% Zn	10% Zn	15% Zn
Cell viability [% of PS control]	100 ± 7.0	35.5 ± 3.9 *	6.4 ± 1.9 *^,#^	47.6 ± 8.2 *^,#,^^	32.7 ± 7.9 *^,^,@^	7.3 ± 2.1 *^,#,@,$^
Ca^2+^ concentration in sample extracts [μg/mL]	68.5 ± 4.8	57.0± 3.9	33.7 ± 5.3 *^,#^	52.0 ± 1.2 *^,^^	58.9 ± 1.7 ^^^	55.6 ± 8.1 ^^^
${\mathrm{HPO}}_{4}^{2-}$ concentration in sample extracts [μg/mL]	43.1 ± 2.8	145.1 ± 1.1 *	179.2 ± 4.2 *^,#^	166.2 ± 2.6 *^,#,^^	163.2 ± 4.7 *^,#,^^	170.7 ± 0.6 *^,#,^^

(*)indicates statistically significant differences between the PS control and other samples, (^#^)indicates statistically significant results between 0% ZnO (-ESM) sample and other samples, (^^^)indicates statistically significant results between 0% ZnO sample and the other samples, (^@^)indicates statistically significant results between 5% ZnO sample and other samples, (^$^)indicates statistically significant results between 10% ZnO sample and other samples, according to one-way ANOVA with post hoc Tukey’s test (*p* < 0.05).

## Data Availability

Not applicable.

## Supplementary Materials

The following supporting information can be downloaded at: https://www.mdpi.com/article/10.3390/ma16051971/s1, Figure S1: Pore size distribution of the obtained materials without Zn (0% Zn) as well as doped with initial 5, 10, 15 and 20 mol % of Zn; Figure S2: TEM image and TEM-EDS profile of the: (a) 0% Zn sample, (b) 10% Zn sample.
