# Supplementary material for: Zn-doped Mono- and Biphasic Calcium Phosphate Materials Derived from Agriculture Waste and Their Potential Biomedical Applications: Part I
Source: Materials (Basel). 2023 Feb 28;16(5):1971. doi: 10.3390/ma16051971 (PMC10003787; doi:10.3390/ma16051971)
Supplement: Supplementary file 1 [file materials-16-01971-s001.zip › materials-2201266-supplementary.pdf]

materials

Article

Zn-Doped Mono- and Biphasic Calcium Phosphate Materials Derived from Agriculture Waste and Their Potential Biomedical Applications: Part I

Marta Kalbarczyk, Aleksandra Szcześ, Anna Belcarz, Paulina Kazimierczak and Zoltan May

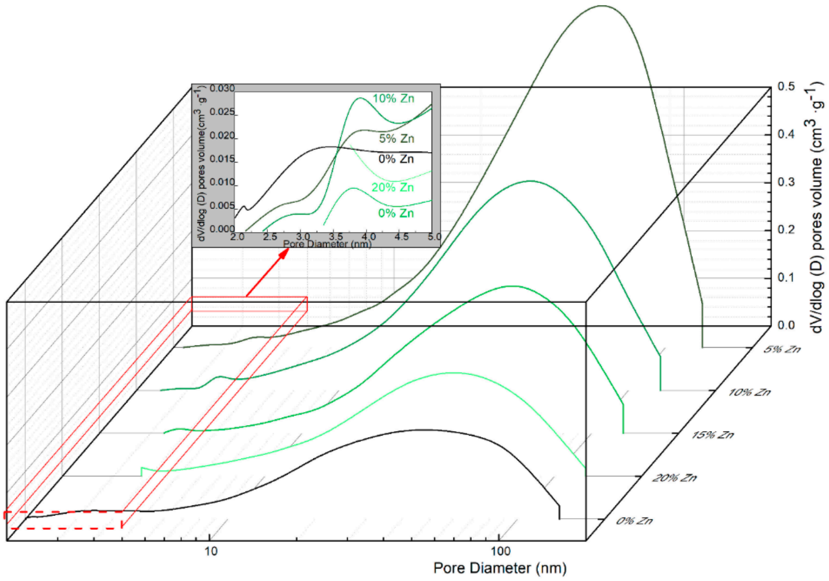

Figure S1. Pore size distribution of the obtained materials without Zn (0% Zn) as well as doped with initial 5, 10, 15 and 20 mol % of Zn.

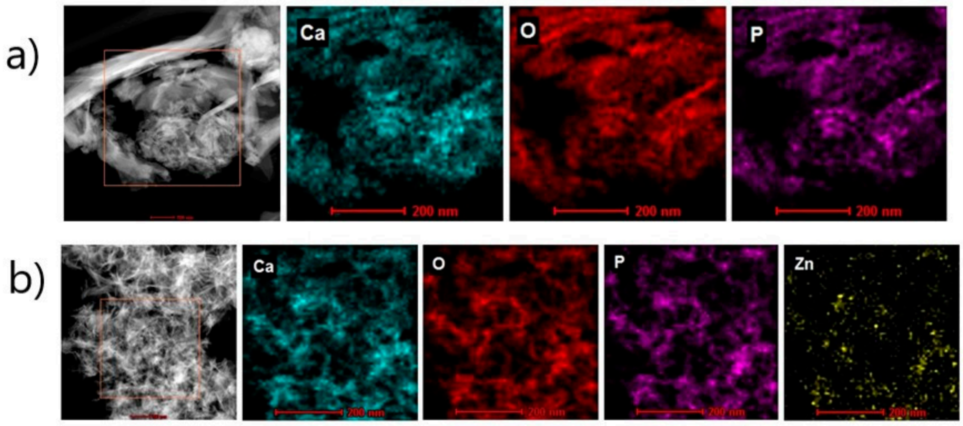

Figure S2. TEM image and TEM-EDS profile of the: a) 0% Zn sample, b) 10% Zn sample.
